# Modulation of Opioid Transport at the Blood-Brain Barrier by Altered ATP-Binding Cassette (ABC) Transporter Expression and Activity

**DOI:** 10.3390/pharmaceutics10040192

**Published:** 2018-10-18

**Authors:** Junzhi Yang, Bianca G. Reilly, Thomas P. Davis, Patrick T. Ronaldson

**Affiliations:** 1 Department of Pharmacology and Toxicology, College of Pharmacy, University of Arizona, 1295 N. Martin St., P.O. Box 210207, Tucson, AZ 85721, USA; jzyang345@email.arizona.edu (J.Y.); davistp@email.arizona.edu (T.P.D.); 2 Department of Pharmacology, College of Medicine, University of Arizona, 1501 N. Campbell Ave, P.O. Box 245050, Tucson, AZ 85724-5050, USA; biancareilly@email.arizona.edu

**Keywords:** pain, opioids, blood-brain barrier, ATP-binding cassette transporters, nuclear receptor signaling, protein trafficking

## Abstract

Opioids are highly effective analgesics that have a serious potential for adverse drug reactions and for development of addiction and tolerance. Since the use of opioids has escalated in recent years, it is increasingly important to understand biological mechanisms that can increase the probability of opioid-associated adverse events occurring in patient populations. This is emphasized by the current opioid epidemic in the United States where opioid analgesics are frequently abused and misused. It has been established that the effectiveness of opioids is maximized when these drugs readily access opioid receptors in the central nervous system (CNS). Indeed, opioid delivery to the brain is significantly influenced by the blood-brain barrier (BBB). In particular, ATP-binding cassette (ABC) transporters that are endogenously expressed at the BBB are critical determinants of CNS opioid penetration. In this review, we will discuss current knowledge on the transport of opioid analgesic drugs by ABC transporters at the BBB. We will also examine how expression and trafficking of ABC transporters can be modified by pain and/or opioid pharmacotherapy, a novel mechanism that can promote opioid-associated adverse drug events and development of addiction and tolerance.

## 1. Introduction

Several currently marketed drugs are known to have pain-relieving properties; however, opioids are the most widely used and the most effective analgesics for moderate to severe pain [1]. Despite their effectiveness as analgesics, opioids are known to cause clinically significant and centrally mediated adverse events including, but not limited to, somnolence, confusion, and respiratory depression. Additionally, these drugs have potential for addiction and for development of tolerance. Despite these clinically significant concerns, overall use of opioids for chronic non-cancer pain has increased in the United States over the past two decades [2]. Furthermore, prescription pain relievers containing opioids are often used for non-medical purposes (i.e., opioid misuse), an established characteristic of the prescription drug abuse problem in the United States [3,4,5]. Indeed, overuse, misuse, and abuse of opioids is a considerable public health concern that requires a detailed understanding of biological mechanisms that can exacerbate adverse events associated with these drugs. Such knowledge is critical to inform development of dosing strategies to counteract misuse of analgesics and to produce safer medications that can be used for acute and chronic non-cancer pain.

Optimal pharmacotherapy with opioids requires the ability to achieve effective concentrations in the brain [6,7,8]. For example, the ability of codeine to provide measurable pain relief requires efficient brain uptake, which is accomplished by passive diffusion [9,10]. Central receptor mediation is also a significant component of the analgesic properties of morphine, oxycodone or hydrocodone [11], which implies that opioids must cross the blood-brain barrier (BBB) and attain effective unbound concentrations in brain tissue. Several BBB mechanisms that determine CNS opioid delivery have been described in the scientific literature [6,12,13,14,15,16,17,18,19,20,21]. Our laboratory has demonstrated the requirement of BBB permeation as a critical determinant of opioid analgesic effectiveness by studying codeine in an in vivo model of acute inflammatory pain [17]. We observed that reduction of codeine brain delivery corresponded to decreased antinociception as measured by paw withdrawal latency from an infrared heat source (i.e., Hargreave’s method) [17]. In addition to therapeutic effectiveness, CNS delivery of opioids across the BBB is a critical factor in the onset of centrally mediated adverse events such as respiratory depression as well as development of addiction and tolerance [8]. Therefore, modifications to BBB integrity (i.e., from pain itself or from drugs administered in conjunction with opioids) can contribute to dramatic changes in CNS levels of opioids, cause changes in opioid analgesic effectiveness, and induce clinically significant adverse events.

Here, we review current literature on ATP-binding cassette (ABC) transporters and their involvement in determining CNS delivery of opioids. Indeed, ABC transporters are primary active transporters that are endogenously expressed at the BBB and greatly impede blood-to-brain transport of many currently marketed opioids. ABC transport proteins at the BBB are also highly dynamic as indicated by changes in their functional expression and trafficking induced by pain or by pharmacotherapy, which can have a significant impact on CNS opioid delivery. Therefore, we will discuss how alterations in ABC transporter expression and/or trafficking constitute a biological mechanism for opioid-associated adverse events and for development of addiction and tolerance. Finally, we will provide perspective on how the understanding of changes in ABC transporters at the BBB can lead to development of novel strategies to provide optimal pain relief and treatment of opioid overuse and/or misuse in the United States.

## 2. Opioid Analgesic Drugs

The cultivation of the opium poppy (*Papaver somniferum*) and the production of opium, a brown, gummy paste dried from the milky juice in the seed pods of the plant, has been documented since the early ages of human civilization [22,23,24,25]. Through centuries of scientific inquiry and decades of pharmacological development, opioids have become one of the major classes of drugs used in modern medicine. The opiate family of compounds is comprised of morphine as well as several other naturally-occurring or semi-synthetic compounds that can be used to alleviate pain, such as heroin, codeine, hydrocodone, oxycodone, and oxymorphone [24,25]. Additionally, there are many synthetic opioids that have been declared illegal due to their abuse potential. These drugs are chemically similar and act on the same molecular targets as the naturally occurring opiates. Fentanyl, meperidine, methadone, and loperamide are among the small fraction of synthetic opioids that have been approved by the FDA and are frequently prescribed for medicinal use [22,23,24,25]. Indeed, opioids are the most widely used and the most effective analgesics for management of moderate to severe pain [1,26,27,28]. Use of opioid analgesics has dramatically increased, as evidenced by a 149% increase in overall opioid prescriptions in the United States from 1997–2007 [26]. Furthermore, Bedson and colleagues reported that, between 2002 and 2013, prescription of “controlled” opioids (i.e., hydrocodone, morphine. oxycodone) increased from 3.5% to 22.6% in a study of patients suffering from chronic musculoskeletal pain [28]. In this same study, it was also observed that over 95% of initial opioid prescriptions included “noncontrolled” opioids such as codeine [28]. Opioids are unique in that they exert their analgesic effect by binding to at least one of three specific pharmacological receptors (i.e., μ-, κ- and δ-opioid receptors) that are localized to neural tissue both within the CNS and in the periphery [29]. The role of these opioid receptors in mediating analgesia as well as opioid-associated reward behaviors and withdrawal symptoms has been extensively demonstrated using genetic knockout models. For example, mice lacking the μ-opioid receptor show no overt behavioral abnormalities or compensatory changes in the opioid system; however, these animals do not experience analgesia, place-preference activity, or physical dependence when administered morphine [30]. Although opioids can exert an analgesic effect by binding to peripheral opioid receptors [31], optimal pharmacotherapy with these drugs requires the ability to access CNS opioid receptors [6,7]. Opioid receptors are G-protein coupled receptors and, therefore, have profound effects on ion gating, intracellular calcium concentrations, and protein phosphorylation [32]. Since there are multiple opioid receptor subtypes, the possibility exists for opioids to exert variable pharmacological effects at different receptors exists. For example, morphine is a full agonist at the μ-opioid receptor but a weak agonist at both the κ- and δ-receptors. In contrast, codeine is a weak agonist at both the μ- and δ-receptors [33,34].

Opioid analgesics have multiple actions in the CNS, not all of which are beneficial. They are known to cause euphoria that, in part, accounts for their abuse potential. Pharmacotherapy with opioids is associated with several adverse events including, but not limited to, somnolence, confusion, respiratory depression and rapid development of tolerance [35]. Indeed, activation of opioid receptors in the brainstem can disrupt respiratory rhythm and cause diminished breathing [36,37]. The role of opioid receptors in controlling respiration is further demonstrated by the fact that direct antagonism of opioid receptors with specific antagonists (i.e., naloxone, naltrexone) can restore respiratory drive in individuals exposed to high doses of opioids [37]. With respect to tolerance, the precise mechanism underlying desensitization of opioid receptors is unknown; however, development of tolerance may involve increased neuronal activity of various protein kinase C isoforms [38,39]. Such adverse opioid effects may limit the opioid dose that can be administered as well as the level of analgesia that can be achieved. Additionally, these adverse events are enhanced by opioid-induced glial activation, which is mediated by the Toll-like receptor (TLR)-4 at the glial cell surface [40]. Activation of glial TLR4 receptors is involved in pathogenesis of neuropathic pain and directly counteracts opioid analgesic efficacy [40]. Although opioid-mediated activation of glial TLR4 signaling can be blocked by co-administration of (+)-naloxone or (+)-naltrexone [41], it remains critical that opioid concentrations in the brain be maintained precisely to ensure efficacious management of pain and limit adverse drug events. This therapeutic objective emphasizes the importance of understanding biological mechanisms at the BBB that are involved in determining CNS delivery of opioid analgesics.

## 3. The Blood-Brain Barrier (BBB)

**Figure 1:**
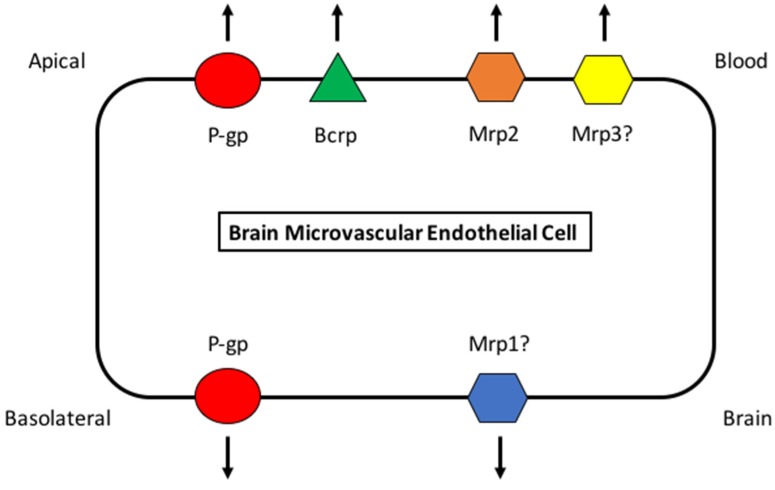
Blood-Brain Barrier Localization of ATP-Binding Cassette (ABC) Transporters relevant to Opioid Transport.

The CNS is the most critical and sensitive organ system in the human body. Proper neuronal and glial function requires precise regulation of the brain extracellular milieu. Therefore, the interface between the CNS and the systemic circulation must be highly selective and must possess effective mechanisms that facilitate nutrient transport, exactly regulate ion balance, and provide a barrier to potentially toxic substances that may be present in the systemic circulation. The BBB performs this integral role by acting as a physical and biochemical barrier between the brain and the systemic circulation. The “physical” barrier properties of the brain microvasculature are conferred by tight junctions that seal the paracellular spaces between adjacent endothelial cells. These tight junctions are comprised of multiprotein complexes of transmembrane proteins (i.e., claudins, occludin, junctional adhesion molecules) that are linked to the actin cytoskeleton by intracellular accessory proteins (i.e., zonulae occludens) [42,43]. The presence of tight junctions requires circulating substances to access the brain via passive transcellular diffusion or to utilize a transport mechanism (i.e., transport proteins or endocytotic processes) for delivery into the brain extracellular milieu. Transporters are critical components of the “biochemical” barrier characteristics of brain microvascular endothelial cells. A plethora of uptake and efflux transport proteins are endogenously expressed at the BBB and allow for selective delivery of solutes into brain parenchyma. For detailed information on the physiology of the BBB, the reader is directed to recent publications from our laboratory [43,44,45]. In general, small (<400 Da), uncharged, lipid-soluble (ClogP ≥ 2.5) compounds with low hydrogen bonding capability (N + O ≤ 5) and minimal affinity for an efflux transport system can easily enter the brain by passive nonionic diffusion. In contrast, CNS uptake of larger, water-soluble, and/or ionic substances is less likely to occur by passive processes. For many compounds, brain penetration is governed by endogenously expressed transport proteins. Many transport proteins that have been shown to be involved in influx and/or efflux of opioid analgesics and/or opioid peptides (i.e., ATP-binding cassette (ABC) transporters, solute carrier (SLC) transporters) have been shown to be selectively expressed at the BBB endothelium [44,46]. Here, we will focus on ABC transporters and their critical role in controlling CNS opioid delivery. Localization of specific ABC transporters known to be determinants of CNS delivery of opioids at the brain microvascular endothelium is depicted in Figure 1. The substrate and/or inhibitor characteristics of opioid analgesics and/or experimental opioid analgesic peptides for various ABC transporters are presented in Table 1.

## 4. ABC Transporters and Opioid Transport at the Blood-Brain Barrier

The ABC superfamily is among the largest and most ubiquitously expressed transporter protein families described to date. ABC transporters directly utilize biological energy generated from ATP hydrolysis to translocate opioids and their metabolites against their concentration gradient (i.e., primary active transport). In humans, 48 ABC genes have been identified and classified according to seven subfamilies [47,48,49]. ABC drug transporters, specifically, P-glycoprotein (P-gp), Multidrug Resistance Proteins (MRPs in humans; Mrps in rodents), and breast cancer resistance protein (BCRP in humans; Bcrp in rodents; also known as ABCG2) are known to be involved in cellular extrusion of therapeutic agents and thus constitute a considerable barrier to effective opioid delivery to the brain. In general, P-gp transports cationic or basic and neutral compounds, whereas MRPs/Mrps are involved in cellular efflux of anionic drugs as well as their glucuronidated, sulfated, and glutathione-conjugated metabolites [44,46]. BCRP/Bcrp has considerable overlap in substrate specificity profile with P-gp and recognizes a vast array of sulfoconjugated organic anions, hydrophobic, and amphiphilic compounds [50]. In fact, BCRP/Bcrp has been shown to function in a synergistic manner with P-gp to limit brain permeation of drugs [51,52,53,54,55].

Perhaps the most critical efflux transport protein for opioids that is expressed at the brain microvascular endothelium is Permeability-glycoprotein (i.e., P-gp). P-gp is a 170 kDa ATP-dependent integral membrane protein that was originally identified in colchicine-resistant Chinese Hamster Ovary (CHO) cells [56]. It was designated as “P-gp” because of its inherent ability to affect permeability of biological membranes to circulating solutes that may potentially be toxic [56]. P-gp orthologues from different species have greater than 70% sequence identity [57] and are encoded by the multidrug resistance (MDR) gene. There are two isoforms of the MDR gene in humans, designated MDR1 and MDR2 while there are three isoforms in both mice and rats (i.e., mdr1a, mdr1b, mdr2). MDR2 and mdr2 gene products are exclusively involved in transport of phosphatidylcholine in the liver. In contrast, human MDR1 and rodent mdr1a/mdr1b are involved in transport of drugs in several tissues including the BBB. P-gp transport susceptibility for currently marketed opioids is variable. Indeed, several opioid analgesic drugs (i.e., morphine, methadone, buprenorphine, fentanyl, loperamide) and associated metabolites (i.e., morphine-6-glucuronide) have been identified as P-gp transport substrates [12,13,15,18,20,58,59,60,61,62,63,64,65]. In contrast, other currently marketed opioids (i.e., codeine, hydrocodone, oxycodone) are not effectively transported by P-gp [64,65,66,67,68,69].

The MRP/Mrp family belongs to the ABC subfamily C (ABCC) group of proteins. At present, nine MRPs have been identified in human tissues that are capable of mediating transport of drugs [70]. Many of the functionally characterized MRP isoforms that are known to be involved in drug transport have been localized to the mammalian BBB. These include MRP1/Mrp1, MRP2/Mrp2, MRP4/Mrp4, Mrp5 and Mrp6 [70,71,72,73,74,75,76,77]. MRP3 has been detected at the mRNA level in cultured human brain endothelial cells [78]; however, its protein expression has only been observed at the brain microvasculature in glioma tissue isolated from cancer patients that also had a diagnosis of epilepsy [79]. Knockdown of Mrp1 by antisense technology in male Sprague-Dawley rats resulted in significant changes to morphine antinociception [80]. Similar results were obtained in Mrp(−/−) mice, which suggests that morphine may be an Mrp1 transport substrate [80]. These observations are particularly compelling in that decreased expression of an ABC efflux transporter caused a reduction in the pain-relieving properties of morphine. This effect may be explained by the proposed BBB localization of Mrp1, which is believed to be expressed at the basolateral plasma membrane on microvascular endothelial cells [76,81]. The ability of some MRP isoforms to transport glucuronidated metabolites may also have considerable consequences for opioid-mediated analgesia. Previous studies in rat hepatocytes [82] and in an in vivo cholestasis model [83] have reported involvement of MRP2 and MRP3 in the efflux of morphine-3-glucuronide. Although morphine-3-glucuronide is known to have minimal agonist activity at opioid receptors [84], it may act as an antagonist to analgesic effects induced by morphine and its active metabolite morphine-6-glucuronide [85]. Therefore, MRP-mediated efflux of morphine-3-glucuronide may be a limiting factor in pharmacological activity of this metabolite.

A third ABC superfamily member that may be involved in drug efflux is BCRP/Bcrp. Several studies have demonstrated localization of BCRP/Bcrp at the BBB, particularly at the luminal plasma membrane of brain microvascular endothelial cells [86,87,88,89,90]. Opioids such as buprenorphine and norbuprenophine have been shown to interact with BCRP at the BBB as inhibitors of drug transport [91]. Further studies are necessary to clarify the functional significance of this transporter at the BBB including the role of BCRP in CNS delivery of opioids.

## 5. Opioids Modulate ABC Transporter Functional Expression at the BBB

Current knowledge in pain pharmacotherapy emphasizes that opioid analgesic drugs can modulate expression of ABC transporters at the BBB. Such effects are important considerations in patients that suffer from non-cancer pain and are prescribed opioids on a chronic basis. Indeed, increased expression and/or activity of efflux transporters by opioids can reduce CNS concentrations and therapeutic effectiveness for these drugs. For example, Yousif and colleagues observed that chronic morphine treatment caused a modest increase in P-gp expression in large cerebral blood vessels isolated from rats [14]. Additionally, P-gp and Bcrp protein levels were shown to be increased 1.5-fold at the BBB in rats subjected to a subchronic morphine treatment paradigm (i.e., 10–40 mg/kg morphine i.p., administered twice daily) [92]. The induction of these ABC transporters was shown to be regulated by an NMDA receptor/cyclo-oxygenase-2 signaling mechanism [92]. P-gp and Bcrp mRNA levels in brain microvessels were also increased in animals subjected to naloxone-precipitated morphine withdrawal [19]. Interestingly, continuous intravenous morphine infusion over a 5-day period had no effect on mRNA levels for P-gp or Bcrp [19]. Similarly, oxycodone has also been shown to increase P-gp functional expression at the BBB, an effect that reduced CNS distribution of P-gp substrate drugs [93]. Taken together, these results indicate that tolerance resulting from chronic exposure to opioids can occur due to enhanced expression of ABC transporters at the BBB, a mechanism that reduces CNS exposure to these drugs. A serious concern is that these observations indicate that patients would require increased/escalating doses of the transporter substrate opioid to overcome the increase in ABC efflux transporter expression at the BBB.

**Figure 2:**
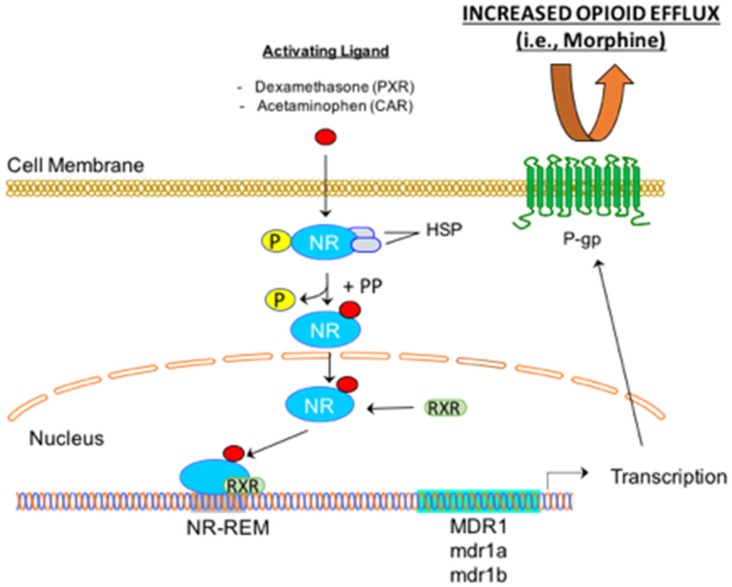
Mechanism of Nuclear Receptor Signaling in Brain Microvascular Endothelial Cells. At the blood-brain barrier, nuclear receptors can be activated by drugs used in the management of pain (i.e., dexamethasone, acetaminophen (APAP)). Once the ligand has bound to the nuclear receptor (NR), heat shock proteins (Hsp) dissociate and the NR is dephosphorylated by a protein phosphatase (PP). This process enables the NR to translocate to the nucleus and bind to its response element (NR-REM) on a target gene. This results in increased expression of ABC transporters such as P-glycoprotein (P-gp) at the endothelial plasma membrane, a mechanism that leads to reduced CNS accumulation of opioids that are transporter substrates.

In addition to opioids, ABC transporters at the BBB are modulated by drugs that are commonly administered concurrently with opioids for management of pain. Such effects are mediated by nuclear receptors, ligand-activated transcription factors that are known to be activated by a broad spectrum of xenobiotics [94]. Some of these receptors, specifically, pregnane-X-receptor (PXR) and constitutive androstane receptor (CAR), are known to be expressed by brain microvascular endothelial cells and are implicated in the regulation of ABC transporters (Figure 2) [18,60,95,96,97,98,99]. The pharmacological impact of PXR-mediated up-regulation of P-gp was demonstrated in a human PXR transgenic mouse model where PXR activation of two specific ligands (i.e., rifampin, hyperforin) reduced CNS uptake of methadone, an established P-gp substrate [60]. Microdialysis studies using male CD-1 mice showed that dexamethasone treatment (5 mg/kg/day for 3 days) resulted in increased brain capillary expression of P-gp and reduced concentrations of the P-gp substrate quinidine in brain extracellular fluid [99]. Furthermore, studies in isolated mouse and rat capillaries showed increased expression and activity of P-gp, Mrp2, and Bcrp when tissues were exposed to 1,4-bis-[2-(3,5-dichloropyridyloxy]benzene (TCPOBOP, a mouse-specific CAR agonist) and phenobarbital (i.e., a rat specific CAR agonist) respectively [98]. These data suggest that PXR/CAR activation may alter the BBB efflux of opioid analgesics by altering functional expression of ABC transporters.

It has also been estimated that uncontrolled activation of nuclear receptors may account for up to 60% of drug-drug interactions [100]. Many drugs included in pain management regimens, particularly anti-inflammatory drugs, have been reported to interact with PXR and/or CAR in both in vitro and in vivo model systems. For example, dexamethasone, an anti-inflammatory glucocorticoid and established PXR activator [101] is often used in the management of pain conditions [102,103]. Dexamethasone has also been shown to reduce antinociceptive effects of opioids such as morphine and β-endorphin [104,105]; however, these studies did not determine if reduced analgesic effects in animals administered dexamethasone involved changes in CNS opioid concentrations. Acetaminophen (APAP) is also commonly prescribed for management of non-cancer pain and is a known CAR activator [18,106]. Our laboratory has demonstrated that APAP (i.e., *N-*acetyl-*p-*aminophenol, paracetamol) increased functional expression of P-gp at the BBB via a CAR-dependent mechanism [18]. This mechanism results in reduced blood-to-brain transport of morphine and reduced analgesic effectiveness of morphine [18]. Taken together, these data suggest that inclusion of anti-inflammatory drugs and/or APAP in pain-treatment regimens can modulate ABC transporters that determine CNS delivery of opioids. Indeed, many patients who are prescribed combination products containing APAP and an opioid for management of moderate to severe non-cancer pain also consume APAP in excess of the maximum daily limit of 4000 mg/day [107]. This is particularly relevant to women because they are more likely to be prescribed prescription pain relievers for treatment of chronic non-cancer pain, to be given higher doses, and to use them for longer periods of time than men [108,109]. Furthermore, there is a disproportionate increase in the misuse of APAP-containing combination opioid products [110]. Such individuals take large doses of APAP, either by itself or in a combination product, in order to achieve euphoria; however, this approach also renders adverse drug events such as respiratory depression more likely. Therefore, activation of nuclear receptors by drugs such as APAP may facilitate clinically significant drug-drug interactions leading to modified opioid analgesia and/or increase the probability of adverse drug events associated with opioid pharmacotherapy.

## 6. Pain Modulates ABC Transporter Expression and Trafficking at the BBB

In addition to pharmacological stimuli, pathophysiological stimuli are well-known to alter the expression of ABC transport proteins at the BBB. Indeed, such effects have been described for P-gp in the context of HIV-1 infection [111,112,113], inflammation [114,115], and epilepsy [116]. Additionally, peripheral inflammatory pain (PIP) is known to modulate ABC transporter functional expression at the BBB. PIP is characterized by release of inflammatory mediators (i.e., TNF-α, IL-1β, IL-6) into the systemic circulation at the site of tissue injury. Cytokines are known to contribute to transduction of acute pain signals by direct activity at nociceptors and via stimulation of prostaglandin release. These nociceptive inputs are then conveyed to the brain through neuronal signaling via the spinal cord. Our laboratory has shown increased protein expression and activity of P-gp at the BBB in rats subjected to λ-carrageenan-induced inflammatory pain (CIP) [12,20,117]. This increase in brain microvascular P-gp expression following CIP corresponded to a decrease in brain uptake of morphine [12]. Kinetically, this decrease in morphine uptake resulted in a 36% decrease in the apparent brain volume of distribution and a 50% increase in the efflux coefficient (k_out_), which reflects a reduction in the amount of morphine that was able to access the brain [12]. This study also showed a reduction in morphine antinociception, an effect that illustrates the critical importance of P-gp in controlling brain opioid uptake and, subsequently, opioid analgesic effectiveness. Furthermore, our results are consistent with the previous work of Hamabe and colleagues who showed that the level of morphine analgesia was inversely proportional to P-gp expression at the BBB [6]. Our studies on morphine antinociception differ from previous work by Ossipov and colleagues that showed increased morphine analgesia following peripheral inflammatory pain [118]. This discrepancy can be explained by sex differences since we used female Sprague-Dawley rats and the study by Ossipov and colleagues utilized male rats. Androgens have been shown to repress transporters including P-gp [119]. This implies that the level of morphine analgesia observed in the Ossipov study may have occurred as a result of a greatly diminished P-gp effect. Nonetheless, the results of all studies with morphine clearly demonstrate that BBB transport mechanisms are critical in determining opioid brain uptake analgesic efficacy.

**Figure 3:**
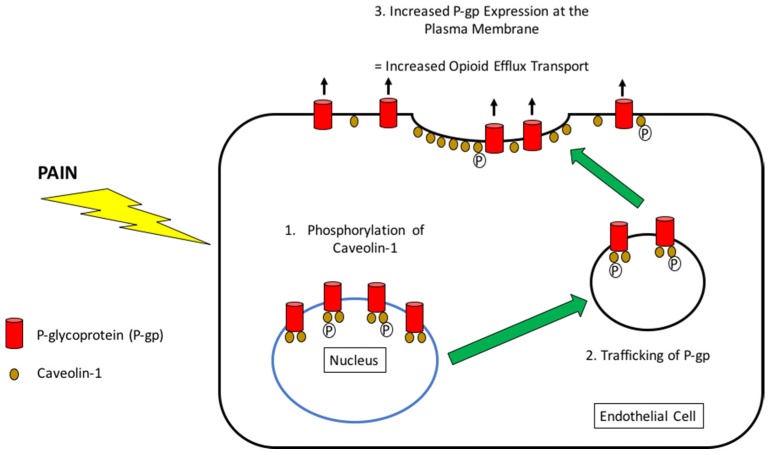
P-glycoprotein Trafficking in Brain Microvascular Endothelial Cells in Response to Acute Inflammatory Pain. Our laboratory has shown caveolin-dependent trafficking of P-glycoprotein (P-gp) from nuclear storage pools to the plasma membrane in the setting of acute inflammatory pain. The events involved in this process are 1. Phosphorylation of caveolin-1, the primary signal triggering recruitment of P-gp to the plasma membrane; 2. Vesicular trafficking of P-gp; and 3. Insertion of P-gp into plasma membrane domains where the transporter is active and able to participate in efflux of substrate drugs such as opioids.

The complexity of P-gp-mediated transport regulation at the BBB in pain is emphasized by knowledge that pain triggers increased P-gp trafficking from the endothelial cell nucleus to the plasma membrane [21,120,121]. Using our detergent-free subcellular fractionation method [109], we demonstrated that P-gp traffics from the nucleus and increases P-gp protein expression and activity at the plasma membrane in response to the CIP stimulus (Figure 3) [120]. Furthermore, these observations suggest that the endothelial cell nucleus acts as a storage pool of P-gp protein that can be rapidly mobilized to the plasma membrane [120]. This is consistent with previous immunogold immunocytochemistry studies that demonstrated P-gp localization at the nuclear envelope in microvascular endothelial cells in human and rat brain [122]. The trafficking mechanism that has been identified and characterized by our group involves phosphorylation of caveolin-1, which is the primary signal that causes enhanced recruitment of P-gp to the plasma membrane in response to acute inflammatory pain [121]. This highly significant finding is supported by our previous work, which demonstrates that P-gp colocalizes with caveolar proteins (i.e., caveolin-1, cavin-1, and cavin-2) in membrane fractions derived from intact brain microvessels [123]. It is important to note that these trafficking events were observed after 3 h CIP, a time point where de novo synthesis of P-gp is unlikely. Of particular importance, P-gp trafficking was enhanced 2.2-fold in female Sprague-Dawley rats that were exposed to morphine for 6 days [21]. The rapid relocalization of P-gp from nuclear storage sites to the plasma membrane in pain or following chronic morphine administration implicates the BBB as a critical site involved in development of opioid tolerance. Indeed, we have shown that increased P-gp expression at the microvascular surface leads to reduced morphine efficacy [12,21]. Therefore, sustained efficacy of morphine (or any other opioid that is also an established P-gp substrate) would require dosage escalations to overcome increased microvascular expression and/or activity that results from P-gp trafficking to the endothelial plasma membrane from nuclear storage pools. Indeed, this process constitutes a “feedback loop” where individuals must consume larger and larger quantities of opioids in order to achieve a desired pharmacological effect. Clearly, P-gp trafficking is an emerging mechanism that must be considered to explain the rapid escalation of opioid abuse and/or misuse in individuals with or without pain.

As noted above, there are several currently marketed opioids that are not substrates for P-gp (i.e., codeine, hydrocodone, oxycodone). This is not to imply that these drugs should be used as alternatives to P-gp substrate opioids in order to limit the potential for opioid-associated adverse events or development of tolerance to opioids. Rather, current knowledge in the BBB field indicates that the physical barrier properties of the brain microvasculature (i.e., protein tight junctions) are altered in response to acute or chronic pain. In particular, our group has shown that CIP promotes measureable modifications to BBB tight junction integrity characterized by dynamic changes in expression and/or trafficking of tight junction proteins (i.e., claudin-5, occludin) and increased paracellular diffusion of solutes present in the circulation [10,17,124,125,126]. These observations are clinically relevant because they can lead to increased brain uptake of opioids such as codeine [10,17]. Loss of BBB integrity demarcated by changes in claudin-5 and/or occludin has also been reported in pain models incorporating chronic spinal nerve ligation [127] and peripheral nerve injury [128], suggesting the BBB changes in experimental in vivo models of pain are not “model-specific” but are critical events involved in the pathophysiological response to pain. Indeed, such molecular changes in the setting of pain can lead to altered CNS drug delivery, opioid effectiveness, and occurrence of opioid-associated adverse events such as respiratory depression and development of tolerance. It is important to point out that brain uptake of P-gp substrate opioids are likely to be more susceptible to P-gp changes and less affected by increased paracellular “leak”. This is supported by our work with morphine, where we reported reduced CNS uptake of this commonly prescribed opioid in animals subjected to CIP [12]. This observation was obtained even though tight junction proteins also change in this in vivo pain model [124,126,129], an effect that can increase paracellular diffusion of non-P-gp substrate drugs such as codeine [10].

## 7. Modulation of ABC Transporter Expression in Other CNS Diseases

In addition to their use in the treatment of acute and chronic pain, opioids have been shown to be effective drugs for other CNS disorders. For example, opioids have potential to be efficacious neuroprotectants [130,131,132,133], which renders them compelling therapeutics for diseases such as ischemic stroke and traumatic brain injury (TBI). Indeed, preclinical studies in rodent models of experimental ischemic stroke (i.e., middle cerebral artery occlusion (MCAO)) have shown that biphalin, a non-selective opioid agonist, can reduce the cerebral infarction area and the brain edema ratio [134,135,136]. Additionally, biphalin was shown to improve neuroscore measurements and locomotor performance 24 h after onset of MCAO, which indicates that this opioid can promote improvement in functional outcomes following experimental ischemic stroke [136]. The direct involvement of opioid receptors in neuroprotection was demonstrated by the observation that the opioid receptor antagonist naltrexone attenuated effects of biphalin on infarction area and edema ratios [136]. Neuroprotective effects in experimental ischemic stroke have been reported for other opioids including the delta opioid receptor agonist D-Ala2-D-Leu2-Enkephalin (DADLE) [137] and the novel multifunctional encephalin-fentanyl opioid agonist LYS739 [138]. The ability of opioids to confer neuroprotection has also been observed in the setting of TBI. For example, a single morphine dose protected against cognitive impairment in mice subjected to mild closed-skull TBI [139]. In this same model, biphalin was also shown to improve performance in the Morris Water Maze and Novel Object Recognition tests, suggesting that this commonly prescribed opioid may also be an effective neuroprotectant [140]. Interestingly, morphine administration did not result in any improvement in motor performance following experimental TBI in adult male rats [141].

The knowledge that opioids may be therapeutic utility as neuroprotectants implies a critical need to understand ABC transporter changes in CNS diseases including ischemic stroke and traumatic brain injury. Such knowledge will facilitate development of opioid-based neuroprotective strategies that can provide optimal therapeutic benefit while also being administered in a safe manner that limits adverse events. Most of the preclinical studies on ABC transporter expression at the BBB is ischemic stroke or TBI have focused on P-gp. Indeed, increased P-gp expression has been reported in cultured rat brain microvessel endothelial cells following oxygen/glucose deprivation (i.e., an in vitro model of ischemia) [142] and at the BBB in experimental animals subjected to MCAO [143,144,145,146]. Interestingly, P-gp protein expression was primarily increased at the luminal membrane of brain microvascular endothelial cells; however, there was no significant change in Mdr mRNA levels, which may imply P-gp trafficking to the plasma membrane post-stroke [143]. Additionally, real-time PCR and quantitative immunohistochemistry studies demonstrated increased Bcrp mRNA and protein in cerebral microvessels at days 3–14 following MCAO [143]. Mrp1 protein levels have been shown to be decreased immediately following MCAO [147]. In contrast, Mrp1 mRNA levels were increased at the BBB in rats subjected to hypoxia/reoxygenation stress, a component of ischemic stroke [77]. Similarly, increased brain microvascular expression of Mrp2 and Mrp4 mRNA was observed in the setting of hypoxia/reoxygenation stress [77]. At present, there is little information regarding ABC transporter expression changes at the BBB following TBI. In one of the few reports that has studied this issue, Pop and colleagues observed long-term persistent decreases in microvascular P-gp expression following controlled cortical impact injury in postnatal day 17 Sprague-Dawley rats [148]. More recently, a study of brain biopsy tissue from patients with severe TBI showed a significant increase in Mrp1 at the BBB but no change in brain microvessel-associated P-gp levels [149]. Indeed, more detailed mechanistic studies (i.e., regulation by signaling pathways and trafficking) are required to rigorously assess the regulation, localization, and functional expression of ABC transporters in CNS diseases such as ischemic stroke and TBI. These studies are all the more critical given the knowledge that many opioids, which have been shown to be provide beneficial effects in the brain such as neuroprotection, are transport substrates for one or more ABC transport proteins.

## 8. Conclusions and Future Perspectives

The field of BBB biology, particularly the study of ABC transporters, has rapidly advanced over the past several years. For example, it is now well established that endogenous efflux transporters such as P-gp are dynamic in nature and can be modified by pathophysiological stimuli (i.e., pain, ischemic stroke, TBI) as well as by pharmacotherapy. These processes can involve rapid relocalization of P-gp to the plasma membrane from storage pools at the endothelial cell nucleus (i.e., trafficking) or activation of specific signaling pathways that can lead to enhancements in transporter functional activity at the BBB. In the context of pain, enhancement of BBB P-gp functional expression by trafficking events or by nuclear receptor pathways can undoubtedly lead to reduced CNS concentrations of opioids that are substrates for ABC transporters. To overcome increased P-gp levels, individuals are likely to respond by consuming higher and higher doses of prescribed or non-prescribed opioids. While this approach can restore analgesic effectiveness, the probability of adverse events and development of addiction and tolerance are also significantly enhanced. Clearly, trafficking events and/or activation of signaling pathways that regulate ABC transporters constitute a novel “BBB-centric” mechanism that has contributed to the current opioid epidemic in the United States.

The specific mechanisms that have been highlighted in this review (i.e., trafficking, nuclear receptor signaling) underscore the need for continued research on alternative strategies to treat pain that do not involve opioids. Some such strategies include, but are not limited to, cannabinoids [150,151], combinations of non-steroidal anti-inflammatory drugs [152], GABA analogs such as pregabalin and gabapentin [153,154], nanoparticle-based delivery of opioid analgesic peptides [155], physical therapy [156], and green light treatment [157]. These approaches are increasing in popularity due to the reduced risk of clinically significant adverse drug events such as respiratory depression as well as decreased probability of drug-seeking behaviors. Despite these advancements, opioids remain medically warranted for many pain conditions and thus are unlikely to become completely absent from prescribed pain pharmacotherapy. This fact indicates a critical need for continued research on biological mechanisms that can modify the potential for opioid-associated adverse events and on new strategies to mitigate these clinically significant risks. While we have focused this review on two important mechanisms that can modulate ABC transporter functional expression at the BBB (i.e., trafficking, nuclear receptor signaling), we appreciate that there may be other “mechanistic triggers” for modulating transporters at the BBB. Future work will continue to describe such mechanisms and provide considerable insight on the role of ABC transporters in determining the CNS effects of substrate opioids. Ultimately, data derived from these studies will enable safer use of opioids as therapeutics as well as development of novel strategies to treat individuals who have abused or misused opioids with the understanding that the BBB plays a central role in opioid pharmacology.

## Figures and Tables

**Table 1:** Known opioid substrates and inducers/inhibitors of ATP-Binding Cassette (ABC) transporters at the blood-brain barrier.

	P-gp	Multidrug Resistance Proteins (MRPs)	Breast Cancer Resistance Protein (BCRP)
***Opioid Analgesic***			
Alfentanil	S		
Buprenorphine	S (−)		(−)
Codeine	N		
Fentanyl	S		
Hydrocodone	N		
Loperamide	S		
Meperidine	S		
Methadone	S (−)		
Morphine	S (+)	S (+)	(+)
(M3G)	S	S	
(M6G)	S	S	
Norbuprenorphine	S		(−)
Oxycodone	N (+)		(+)
Oxymorphone		S	
Sufentanil	N		
Tramadol	S		
***Opioid Peptide***			
Biphalin	N		
Deltorphin II	S		
[D-Ala^2^, N-MePhe^4^, Gly-ol]-enkephalin (DAMGO)	S		
[D-Pen^2^,D-Pen^5^]enkephalin (DPDPE)	S		
β-endorphin	S		
Leu-enkephalin	N		
Met-enkephalin	N		

S: substrate of the transporter; N: known non-substrate of the transporter; (+): inducer of the transporter; (−): inhibitor of the transporter; M3G: morphine-3-glucuronide; M6G: morphine-6-glucuronide.

## References

[1] Chou R., Fanciullo G.J., Fine P.G., Adler J.A., Ballantyne J.C., Davies P., Donovan M.I., Fishbain D.A., Foley K.M., Fudin J. (2009). Clinical Guidelines for the use of Chronic Opioid Therapy in Chronic Noncancer Pain. J. Pain.

[2] Kaye A.D., Jones M.R., Kaye A.M., Ripoll J.G., Galan V., Beakley B.D., Calixto F., Bolden J.L., Urman R.D., Manchikanti L. (2017). Prescription Opioid Abuse in Chronic Pain: An Updated Review of Opioid Abuse Predictors and Strategies to Curb Opioid Abuse: Part 1. Pain Physician.

[3] Shei A., Rice J.B., Kirson N.Y., Bodnar K., Birnbaum H.G., Holly P., Ben-Joseph R. (2015). Sources of Prescription Opioids among Diagnosed Opioid Abusers. Curr. Med. Res. Opin..

[4] Vowles K.E., McEntee M.L., Julnes P.S., Frohe T., Ney J.P., van der Goes D.N. (2015). Rates of Opioid Misuse, Abuse, and Addiction in Chronic Pain: A Systematic Review and Data Synthesis. Pain.

[5] Maree R.D., Marcum Z.A., Saghafi E., Weiner D.K., Karp J.F. (2014). A Systematic Review of Opioid and Benzodiazepine Misuse in Older Adults. Am. J. Geriatr. Psychiatry.

[6] Hamabe W., Maeda T., Kiguchi N., Yamamoto C., Tokuyama S., Kishioka S. (2007). Negative Relationship between Morphine Analgesia and P-Glycoprotein Expression Levels in the Brain. J. Pharm. Sci..

[7] Labuz D., Mousa S.A., Schäfer M., Stein C., Machelska H. (2007). Relative Contribution of Peripheral Versus Central Opioid Receptors to Antinociception. Brain Res..

[8] Schaefer C.P., Tome M.E., Davis T.P. (2017). The Opioid Epidemic: A Central Role for the Blood Brain Barrier in Opioid Analgesia and Abuse. Fluids Barriers CNS.

[9] Xie R., Hammarlund-Udenaes M. (1998). Blood-Brain Barrier Equilibration of Codeine in Rats Studied with Microdialysis. Pharm. Res..

[10] Hau V.S., Huber J.D., Campos C.R., Davis R.T., Davis T.P. (2004). Effect of λ-Carrageenan-Induced Inflammatory Pain on Brain Uptake of Codeine and Antinociception. Brain Res..

[11] Peckham E.M., Traynor J.R. (2006). Comparison of the Antinociceptive Response to Morphine and Morphine-Like Compounds in Male and Female Sprague-Dawley Rats. J. Pharmacol. Exp. Ther..

[12] Seelbach M.J., Brooks T.A., Egleton R.D., Davis T.P. (2007). Peripheral Inflammatory Hyperalgesia Modulates Morphine Delivery to the Brain: A Role for P-Glycoprotein. J. Neurochem..

[13] Suzuki T., Zaima C., Moriki Y., Fukami T., Tomono K. (2007). P-Glycoprotein Mediates Brain-to-Blood Efflux Transport of Buprenorphine across the Blood-Brain Barrier. J. Drug Target..

[14] Yousif S., Saubaméa B., Cisternino S., Marie-Claire C., Dauchy S., Scherrmann J., Declèves X. (2008). Effect of Chronic Exposure to Morphine on the Rat Blood–brain Barrier: Focus on the P-glycoprotein. J. Neurochem..

[15] Hawkins B.T., Sykes D.B., Miller D.S. (2010). Rapid, Reversible Modulation of Blood-Brain Barrier P-Glycoprotein Transport Activity by Vascular Endothelial Growth Factor. J. Neurosci..

[16] Ronaldson P.T., Finch J.D., Demarco K.M., Quigley C.E., Davis T.P. (2011). Inflammatory Pain Signals an Increase in Functional Expression of Organic Anion Transporting Polypeptide 1a4 at the Blood-Brain Barrier. J. Pharmacol. Exp. Ther..

[17] Lochhead J.J., McCaffrey G., Sanchez-Covarrubias L., Finch J.D., DeMarco K.M., Quigley C.E., Davis T.P., Ronaldson P.T. (2012). Tempol Modulates Changes in Xenobiotic Permeability and Occludin Oligomeric Assemblies at the Blood Brain Barrier during Inflammatory Pain. Am. J. Physiol. Heart Circ. Physiol..

[18] Slosky L.M., Thompson B.J., Sanchez-Covarrubias L., Zhang Y., Laracuente M., Vanderah T.W., Ronaldson P.T., Davis T.P. (2013). Acetaminophen Modulates P-Glycoprotein Functional Expression at the Blood-Brain Barrier by a Constitutive Androstane Receptor-Dependent Mechanism. Mol. Pharmacol..

[19] Chaves C., Gómez-Zepeda D., Auvity S., Menet M., Crété D., Labat L., Remião F., Cisternino S., Declèves X. (2016). Effect of Subchronic Intravenous Morphine Infusion and Naloxone-Precipitated Morphine Withdrawal on P-Gp and Bcrp at the Rat Bloode-Brain Barrier. J. Pharm. Sci..

[20] Sanchez-Covarrubias L., Slosky L.M., Thompson B.J., Zhang Y., Laracuente M., DeMarco K.M., Ronaldson P.T., Davis T.P. (2014). P-Glycoprotein Modulates Morphine Uptake into the CNS: A Role for the Non-Steroidal Anti-Inflammatory Drug Diclofenac. PLoS ONE.

[21] Schaefer C.P., Arkwright N.B., Jacobs L.M., Jarvis C.K., Hunn K.C., Largent-Milnes T.M., Tome M.E., Davis T.P. (2018). Chronic Morphine Exposure Potentiates P-Glycoprotein Trafficking from Nuclear Reservoirs in Cortical Rat Brain Microvessels. PLoS ONE.

[22] Schiff P.L. (1980). Opium and its Alkaloids. J. Pharm. Sci..

[23] Brownstein M.J. (1993). A Brief History of Opiates, Opioid Peptides, and Opioid Receptors. Proc. Natl. Acad. Sci. USA.

[24] Manchikanti L., Singh A. (2008). Therapeutic Opioids: A Ten-Year Perspective on the Complexities and Complications of the Escalating use, Abuse, and Nonmedical use of Opioids. Pain Physician.

[25] Gahlinger P.M. (2004). Illegal Drugs: A Complete Guide to Their History, Chemistry, Use and Abuse.

[26] Manchikanti L., Benyamin R., Datta S., Vallejo R., Smith H. (2010). Opioids in Chronic Noncancer Pain. Expert Rev. Neurother..

[27] Gloth F.M. (2011). Pharmacological Management of Persistent Pain in Older Persons: Focus on Opioids and Nonopioids. J. Pain.

[28] Bedson J., Chen Y., Hayward R.A., Ashworth J., Walters K., Dunn K.M., Jordan K.P. (2016). Trends in Long-Term Opioid Prescribing in Primary Care Patients with Musculoskeletal Conditions: An Observational Database Study. Pain.

[29] Martin W.R., Eades C.G., Thompson J.A., Huppler R.E., Gilbert P.E. (1976). The Effects of Morphine- and Nalorphine-Like Drugs in the Nondependent and Morphine-Dependent Chronic Spinal Dog. J. Pharmacol. Exp. Ther..

[30] Matthes H.W., Maldonado R., Simonin F., Valverde O., Slowe S., Kitchen I., Befort K., Dierich A., Le Meur M., Dollé P. (1996). Loss of Morphine Induced Analgesia, Reward Effect and Withdrawal Symptoms in Mice Lacking the Mu Opioid Receptor Gene. Nature.

[31] Vadivelu N., Mitra S., Hines R.L. (2011). Peripheral Opioid Receptor Agonists for Analgesia: A Comprehensive Review. J. Opioid Manag..

[32] Shang Y., Filizola M. (2015). Opioid Receptors: Structural and Mechanistic Insights into Pharmacology and Signaling. Eur. J. Pharmacol..

[33] Sternini C., Spann M., Anton B., Keith D.E., Bunnett N.W., von Zastrow M., Evans C., Brecha N.C. (1996). Agonist-Selective Endocytosis of Mu-Opioid Receptor by Neurons in Vivo. Proc. Natl. Acad. Sci. USA.

[34] Coller J., Christrup L., Somogyi A. (2009). Role of Active Metabolites in the Use of Opioids. Eur. J. Clin. Pharmacol..

[35] Haas D.A. (2002). An Update on Analgesics for the Management of Acute Postoperative Dental Pain. J. Can. Dent. Assoc..

[36] Chevillard L., Megarbane B., Baud F.J., Risede P., Decleves X., Mager D., Milan N., Ricordel I. (2010). Mechanisms of Respiratory Insufficiency Induced by Methadone Overdose in Rats. Addict. Biol..

[37] Olofsen E., van Dorp E., Teppema L., Aarts L., Smith T.W., Dahan A., Sarton E. (2010). Naloxone Reversal of Morphine- and Morphine-6-Glucuronide-Induced Respiratory Depression in Healthy Volunteers: A Mechanism-Based Pharmacokinetic-Pharmacodynamic Modeling Study. Anesthesiology.

[38] Halls M.L., Yeatman H.R., Nowell C.J., Thompson G.L., Gondin A.B., Civciristov S., Bunnett N.W., Lambert N.A., Poole D.P., Canals M. (2016). Plasma Membrane Localization of the Μ-Opioid Receptor Controls Spatiotemporal Signaling. Sci. Signal.

[39] Withey S.L., Hill R., Lyndon A., Dewey W.L., Kelly E., Henderson G. (2017). Effect of Tamoxifen and Brain-Penetrant Protein Kinase C and C-Jun N-Terminal Kinase Inhibitors on Tolerance to Opioid-Induced Respiratory Depression in Mice. J. Pharmacol. Exp. Ther..

[40] Watkins L.R., Hutchinson M.R., Rice K.C., Maier S.F. (2009). The “Toll” of Opioid-Induced Glial Activation: Improving the Clinical Efficacy of Opioids by Targeting Glia. Trends Pharmacol. Sci..

[41] Hutchinson M.R., Zhang Y., Brown K., Coats B.D., Shridhar M., Sholar P.W., Patel S.J., Crysdale N.Y., Harrison J.A., Maier S.F. (2008). Non-stereoselective Reversal of Neuropathic Pain by Naloxone and Naltrexone: Involvement of Toll-like Receptor 4 (TLR4). Eur. J. Neurosci..

[42] Lochhead J.J., Ronaldson P.T., Davis T.P. (2017). Hypoxic Stress and Inflammatory Pain Disrupt Blood-Brain Barrier Tight Junctions: Implications for Drug Delivery to the Central Nervous System. AAPS J..

[43] Abdullahi W., Tripathi D., Ronaldson P.T. (2018). Blood-Brain Barrier Dysfunction in Ischemic Stroke: Targeting Tight Junctions and Transporters for Vascular Protection. Am. J. Physiol. Cell Physiol..

[44] Ronaldson P.T., Davis T.P. (2011). Targeting Blood-Brain Barrier Changes during Inflammatory Pain: An Opportunity for Optimizing CNS Drug Delivery. Ther. Deliv..

[45] Ronaldson P.T., Davis T.P. (2012). Blood-Brain Barrier Integrity and Glial Support: Mechanisms that can be Targeted for Novel Therapeutic Approaches in Stroke. Curr. Pharm. Des..

[46] Sanchez-Covarrubias L., Slosky L.M., Thompson B.J., Davis T.P., Ronaldson P.T. (2014). Transporters at CNS Barrier Sites: Obstacles or Opportunities for Drug Delivery?. Curr. Pharm. Des..

[47] Leslie E.M., Deeley R.G., Cole S.P.C. (2005). Multidrug Resistance Proteins: Role of P-Glycoprotein, MRP1, MRP2, and BCRP (ABCG2) in Tissue Defense. Toxicol. Appl. Pharmacol..

[48] Chaves C., Shawahna R., Jacob A., Scherrmann J., Declèves X. (2014). Human ABC Transporters at Blood-CNS Interfaces as Determinants of CNS Drug Penetration. Curr. Pharm. Des..

[49] Morris M., Rodriguez-Cruz V., Felmlee M. (2017). SLC and ABC Transporters: Expression, Localization, and Species Differences at the Blood-Brain and the Blood-Cerebrospinal Fluid Barriers. AAPS J..

[50] Robey R.W., To K.K.K., Polgar O., Dohse M., Fetsch P., Dean M., Bates S.E. (2009). ABCG2: A Perspective. Adv. Drug Deliv. Rev..

[51] De Vries N.A., Zhao J., Kroon E., Buckle T., Beijnen J.H., van Tellingen O. (2007). P-Glycoprotein and Breast Cancer Resistance Protein: Two Dominant Transporters Working Together in Limiting the Brain Penetration of Topotecan. Clin. Cancer Res..

[52] Polli J.W., Olson K.L., Chism J.P., John-Williams L.S., Yeager R.L., Woodard S.M., Otto V., Castellino S., Demby V.E. (2009). An Unexpected Synergist Role of P-Glycoprotein and Breast Cancer Resistance Protein on the Central Nervous System Penetration of the Tyrosine Kinase Inhibitor Lapatinib (N-{3-Chloro-4-[(3-Fluorobenzyl)Oxy]Phenyl}-6-[5-({[2-(Methylsulfonyl)Ethyl]Amino}Methyl)-2-Furyl]-4-Quinazolinamine; GW572016). Drug Metab. Dispos..

[53] Agarwal S., Elmquist W.F. (2012). Insight into the Cooperation of P-Glycoprotein (ABCB1) and Breast Cancer Resistance Protein (ABCG2) at the Blood–Brain Barrier: A Case Study Examining Sorafenib Efflux Clearance. Mol. Pharm..

[54] Oberoi R.K., Mittapalli R.K., Elmquist W.F. (2013). Pharmacokinetic Assessment of Efflux Transport in Sunitinib Distribution to the Brain. J. Pharmacol. Exp. Ther..

[55] Sane R., Agarwal S., Mittapalli R.K., Elmquist W.F. (2013). Saturable Active Efflux by P-Glycoprotein and Breast Cancer Resistance Protein at the Blood-Brain Barrier Leads to Nonlinear Distribution of Elacridar to the Central Nervous System. J. Pharmacol. Exp. Ther..

[56] Juliano R.L., Ling V. (1976). A surface glycoprotein modulating drug permeability in Chinese hamster ovary cell mutants. Biochim. Biophys. Acta.

[57] Gottesman M.M., Hrycyna C.A., Schoenlein P.V., Germann U.A., Pastan I. (1995). Genetic Analysis of the Multidrug Transporter. Annu. Rev. Genet..

[58] Letrent S.P., Pollack G.M., Brouwer K.R., Brouwer K.L. (1998). Effect of GF120918, a Potent P Glycoprotein Inhibitor, on Morphine Pharmacokinetics and Pharmacodynamics in the Rat. Pharm. Res..

[59] Lötsch J., Schmidt R., Vetter G., Schmidt H., Niederberger E., Geisslinger G., Tegeder I. (2002). Increased CNS Uptake and Enhanced Antinociception of Morphine-6-glucuronide in Rats After Inhibition of P-glycoprotein. J. Neurochem..

[60] Bauer B., Yang X., Hartz A.M., Olson E.R., Zhao R., Kalvass J.C., Pollack G.M., Miller D.S. (2006). In Vivo Activation of Human Pregnane X Receptor Tightens the Blood-Brain Barrier to Methadone through P-Glycoprotein Up-Regulation. Mol. Pharmacol..

[61] Mercer S.L., Coop A. (2011). Opioid Analgesics and P-Glycoprotein Efflux Transporters: A Potential Systems-Level Contribution to Analgesic Tolerance. Curr. Top. Med. Chem..

[62] Tournier N., Declèves X., Saubaméa B., Scherrmann J.M., Cisternino S. (2011). Opioid Transport by ATP-Binding Cassette Transporters at the Blood-Brain Barrier: Implications for Neuropsychopharmacology. Curr. Pharm. Des..

[63] De Gregori S., De Gregori M., Ranzani G., Allegri M., Minella C., Regazzi M. (2012). Morphine Metabolism, Transport and Brain Disposition. Metab. Brain Dis..

[64] Yu C., Yuan M., Yang H., Zhuang X., Li H. (2018). P-Glycoprotein on Blood-Brain Barrier Plays a Vital Role in Fentanyl Brain Exposure and Respiratory Toxicity in Rats. Toxicol. Sci. Toxicol..

[65] Hsiao P., Unadkat J.D. (2012). P-Glycoprotein-Based Loperamide-Cyclosporine Drug Interaction at the Rat Blood-Brain Barrier: Prediction from in Vitro Studies and Extrapolation to Humans. Mol. Pharm..

[66] Boström E., Simonsson U.S.H., Hammarlund-Udenaes M. (2005). Oxycodone Pharmacokinetics and Pharmacodynamics in the Rat in the Presence of the P-Glycoprotein Inhibitor PSC833. J. Pharm. Sci..

[67] Somogyi A., Coller J., Barratt D. (2015). Pharmacogenetics of Opioid Response. Clin. Pharmacol. Ther..

[68] Cunningham C.W., Mercer S.L., Hassan H.E., Traynor J.R., Eddington N.D., Coop A. (2008). Opioids and Efflux Transporters. Part 2: P-Glycoprotein Substrate Activity of 3- and 6-Substituted Morphine Analogs. J. Med. Chem..

[69] Sadiq M.W., Uchida Y., Hoshi Y., Tachikawa M., Terasaki T., Hammarlund-Udenaes M. (2015). Validation of a P-Glycoprotein (P-Gp) Humanized Mouse Model by Integrating Selective Absolute Quantification of Human MDR1, Mouse Mdr1a and Mdr1b Protein Expressions with in Vivo Functional Analysis for Blood-Brain Barrier Transport. PLoS ONE.

[70] Zhang Y., Wang Y., Gupta P., Chen Z. (2015). Multidrug Resistance Proteins (MRPs) and Cancer Therapy. AAPS J..

[71] Miller D.S., Nobmann S.N., Gutmann H., Toeroek M., Drewe J., Fricker G. (2000). Xenobiotic Transport Across Isolated Brain Microvessels Studied by Confocal Microscopy. Mol. Pharmacol..

[72] Leggas M., Adachi M., Scheffer G.L., Sun D., Wielinga P., Du G., Mercer K.E., Zhuang Y., Panetta J.C., Johnston B. (2004). Mrp4 Confers Resistance to Topotecan and Protects the Brain from Chemotherapy. Mol. Cell. Biol..

[73] Zhang Y., Schuetz J.D., Elmquist W.F., Miller D.W. (2004). Plasma Membrane Localization of Multidrug Resistance-Associated Protein Homologs in Brain Capillary Endothelial Cells. J. Pharmacol. Exp. Ther..

[74] Bandler P.E., Westlake C.J., Grant C.E., Cole S.P.C., Deeley R.G. (2008). Identification of Regions Required for Apical Membrane Localization of Human Multidrug Resistance Protein 2. Mol. Pharmacol..

[75] Bauer B., Hartz A.M.S., Lucking J.R., Yang X., Pollack G.M., Miller D.S. (2008). Coordinated Nuclear Receptor Regulation of the Efflux Transporter, Mrp2, and the Phase-II Metabolizing Enzyme, GST, at the Blood-Brain Barrier. J. Cereb. Blood Flow Metab..

[76] Uchida Y., Ohtsuki S., Katsukura Y., Ikeda C., Suzuki T., Kamiie J., Terasaki T. (2011). Quantitative Targeted Absolute Proteomics of Human Blood–brain Barrier Transporters and Receptors. J. Neurochem..

[77] Ibbotson K., Yell J., Ronaldson P.T. (2017). Nrf2 Signaling Increases Expression of ATP-Binding Cassette Subfamily C mRNA Transcripts at the Blood–brain Barrier Following Hypoxia-Reoxygenation Stress. Fluids Barriers CNS.

[78] Dauchy S., Miller F., Couraud P., Weaver R.J., Weksler B., Romero I., Scherrmann J., De Waziers I., Declèves X. (2009). Expression and Transcriptional Regulation of ABC Transporters and Cytochromes P450 in hCMEC/D3 Human Cerebral Microvascular Endothelial Cells. Biochem. Pharmacol..

[79] Calatozzolo C., Pollo B., Botturi A., Dinapoli L., Carosi M., Salmaggi A., Maschio M. (2012). Multidrug Resistance Proteins Expression in Glioma Patients with Epilepsy. J. Neurooncol..

[80] Su W., Pasternak G.W. (2013). The Role of Multidrug Resistance-associated Protein in the Blood–brain Barrier and Opioid Analgesia. Synapse.

[81] Roberts L.M., Black D.S., Raman C., Woodford K., Zhou M., Haggerty J.E., Yan A.T., Cwirla S.E., Grindstaff K.K. (2008). Subcellular Localization of Transporters Along the Rat Blood–brain Barrier and Blood–cerebral-Spinal Fluid Barrier by in Vivo Biotinylation. Neuroscience.

[82] Van de Wetering K., Zelcer N., Kuil A., Feddema W., Hillebrand M., Vlaming M.L.H., Schinkel A.H., Beijnen J.H., Borst P. (2007). Multidrug Resistance Proteins 2 and 3 Provide Alternative Routes for Hepatic Excretion of Morphine-Glucuronides. Mol. Pharmacol..

[83] Hasegawa Y., Kishimoto S., Takahashi H., Inotsume N., Takeuchi Y., Fukushima S. (2009). Altered Expression of MRP2, MRP3 and UGT2B1 in the Liver Affects the Disposition of Morphine and its Glucuronide Conjugate in a Rat Model of Cholestasis. J. Pharm. Pharmacol..

[84] Penson R.T., Joel S.P., Gloyne A., Clark S., Slevin M.L. (2005). Morphine Analgesia in Cancer Pain: Role of the Glucuronides. J. Opioid Manag..

[85] Penson R.T., Joel S.P., Bakhshi K., Clark S.J., Langford R.M., Slevin M.L. (2000). Randomized Placebo-Controlled Trial of the Activity of the Morphine Glucuronides. Clin. Pharmacol. Ther..

[86] Cooray H., Blackmore C., Maskell L., Barrand M. (2002). Localisation of Breast Cancer Resistance Protein in Microvessel Endothelium of Human Brain. Neuroreport.

[87] Hori S., Ohtsuki S., Hosoya K., Nakashima E., Terasaki T. (2004). A Pericyte-derived Angiopoietin-1 Multimeric Complex Induces Occludin Gene Expression in Brain Capillary Endothelial Cells through Tie-2 Activation in Vitro. J. Neurochem..

[88] Ohtsuki S., Ikeda C., Uchida Y., Sakamoto Y., Miller F., Glacial F., Decleves X., Scherrmann J., Couraud P., Kubo Y. (2013). Quantitative Targeted Absolute Proteomic Analysis of Transporters, Receptors and Junction Proteins for Validation of Human Cerebral Microvascular Endothelial Cell Line hCMEC/D3 as a Human Blood-Brain Barrier Model. Mol. Pharm..

[89] Hoque M.T., Shah A., More V., Miller D.S., Bendayan R. (2015). In Vivo and Ex Vivo Regulation of Breast Cancer Resistant Protein (Bcrp) by Peroxisome Proliferator-Activated Receptor Alpha (Pparα) at the Blood–brain Barrier. J. Neurochem..

[90] Bakhsheshian J., Wei B., Hall M.D., Simpson R.M., Gottesman M.M. (2016). In Vivo Bioluminescent Imaging of ATP-Binding Cassette Transporter-Mediated Efflux at the Blood–Brain Barrier. Methods Mol. Biol..

[91] Tournier N., Chevillard L., Megarbane B., Pirnay S., Scherrmann J., Declèves X. (2010). Interaction of Drugs of Abuse and Maintenance Treatments with Human P-Glycoprotein (ABCB1) and Breast Cancer Resistance Protein (ABCG2). Int. J. Neuropsychopharmacol..

[92] Yousif S., Chaves C., Potin S., Margaill I., Scherrmann J.M., Decleves X. (2012). Induction of P-glycoprotein and Bcrp at the rat blood-brain barrier following a subchronic morphine treatment is mediated through NMDA/COX-2 activation. J. Neurochem..

[93] Hassan H.E., Myers A.L., Lee I.J., Coop A., Eddington N.D. (2007). Oxycodone Induces Overexpression of P-glycoprotein (ABCB1) and Affects Paclitaxel’s Tissue Distribution in Sprague Dawley Rats. J. Pharm. Sci..

[94] Kullak-Ublick G.A., Becker M.B. (2003). Regulation of Drug and Bile Salt Transporters in Liver and Intestine. Drug Metab. Rev..

[95] Bauer B., Hartz A.M.S., Fricker G., Miller D.S. (2004). Pregnane X Receptor Up-Regulation of P-Glycoprotein Expression and Transport Function at the Blood-Brain Barrier. Mol. Pharmacol..

[96] Lombardo L., Pellitteri R., Balazy M., Cardile V. (2008). Induction of Nuclear Receptors and Drug Resistance in the Brain Microvascular Endothelial Cells Treated with Antiepileptic Drugs. Curr. Neurovasc. Res..

[97] Narang V.S., Fraga C., Kumar A., Shen J., Throm S., Stewart C.F., Waters C.M. (2008). Dexamethasone Increases Expression and Activity of Multidrug Resistance Transporters at the Rat Blood Brain Barrier. Am. J. Physiol. Cell Physiol..

[98] Wang X., Sykes D.B., Miller D.S. (2010). Constitutive Androstane Receptor-Mediated Up-Regulation of ATP-Driven Xenobiotic Efflux Transporters at the Blood-Brain Barrier. Mol. Pharmacol..

[99] Chan G.N.Y., Saldivia V., Yang Y., Pang H., Lannoy I., Bendayan R. (2013). In Vivo Induction of P-Glycoprotein Expression at the Mouse Blood–Brain Barrier: An Intracerebral Microdialysis Study. J. Neurochem..

[100] Urquhart B., Tirona R., Kim R. (2007). Nuclear Receptors and the Regulation of Drug-Metabolizing Enzymes and Drug Transporters: Implications for Interindividual Variability in Response to Drugs. J. Clin. Pharmacol..

[101] Matheny C.J., Ali R.Y., Yang X., Pollack G.M. (2004). Effect of Prototypical Inducing Agents on P-Glycoprotein and CYP3A Expression in Mouse Tissues. Drug Metab. Dispos..

[102] Stein A., Stein C., Yassouridis A., Szopko C., Helmke K. (1999). Intraarticular Morphine versus Dexamethasone in Chronic Arthritis. Pain.

[103] Kardash K.J., Sarrazin F., Tessler M.J., Velly A.M. (2008). Single-Dose Dexamethasone Reduces Dynamic Pain after Total Hip Arthroplasty. Anesth. Analg..

[104] Pieretti S., Giannuario A.D., Domenici M.R., Sagratella S., Capasso A., Sorrentino L., Loizzo A. (1994). Dexamethasone-Induced Selective Inhibition of the Central It Opioid Receptor: Functional in Vivo and in Vitro Evidence in Rodents. Br. J. Pharmacol..

[105] Capasso A., Loizzo A. (2008). Functional Interference of Dexamethasone on Some Morphine Effects: Hypothesis for the Steroid-Opioid Interaction. Recent Pat. CNS Drug Discov..

[106] Zhang J., Huang W., Chua S.S., Wei P., Moore D.D. (2002). Modulation of Acetaminophen-Induced Hepatotoxicity by the Xenobiotic Receptor CAR. Science.

[107] Hoban B., Larance B., Gisev N., Nielsen S., Cohen M., Bruno R., Shand F., Lintzeris N., Hall W., Farrell M. (2015). The use of Paracetamol (Acetaminophen) among a Community Sample of People with Chronic Non-cancer Pain Prescribed Opioids. Int. J. Clin. Pract..

[108] Paulozzi L.J., Strickler G.K., Kreiner P.W., Koris C.M., Centers for Disease Control and Prevention (CDC) (2015). Controlled Substance Prescribing Patterns—Prescription Behavior Surveillance System, Eight States, 2013. MMWR Surveill. Summ..

[109] Koons A.L., Rayl Greenberg M., Cannon R.D., Beauchamp G.A. (2018). Women and the Experience of Pain and Opioid Use Disorder: A Literature-Based Commentary. Clin. Ther..

[110] Bond G., Ho M., Woodward R. (2012). Trends in Hepatic Injury Associated with Unintentional Overdose of Paracetamol (Acetaminophen) in Products with and without Opioid. Drug Saf..

[111] Hayashi K., Pu H., Tian J., Andras I.E., Lee Y.W., Hennig B., Toborek M. (2005). HIV-Tat Protein Induces P-glycoprotein Expression in Brain Microvascular Endothelial Cells. J. Neurochem..

[112] Ashraf T., Ronaldson P.T., Persidsky Y., Bendayan R. (2011). Regulation of P-glycoprotein by Human Immunodeficiency Virus-1 in Primary Cultures of Human Fetal Astrocytes. J. Neurosci. Res..

[113] Robillard K.R., Hoque M.T., Bendayan R. (2014). Expression of ATP-Binding Cassette Membrane Transporters in a HIV-1 Transgenic Rat Model. Biochem. Biophys. Res. Commun..

[114] Hartz A.M.S., Bauer B., Fricker G., Miller D.S. (2007). Rapid Modulation of P-Glycoprotein-Mediated Transport at the Blood-Brain Barrier by Tumor Necrosis Factor- and Lipopolysaccharide. Mol. Pharm..

[115] Salkeni M., Lynch J., Otamis-Price T., Banks W. (2009). Lipopolysaccharide Impairs Blood–Brain Barrier P-Glycoprotein Function in Mice through Prostaglandin- and Nitric Oxide-Independent Pathways. J. Neuroimmune Pharmacol..

[116] Uchida Y., Ohtsuki S., Terasaki T. (2014). Pharmacoproteomics-Based Reconstruction of in Vivo P-Glycoprotein Function at Blood-Brain Barrier and Brain Distribution of Substrate Verapamil in Pentylenetetrazole-Kindled Epilepsy, Spontaneous Epilepsy, and Phenytoin Treatment Models. Drug Metab. Dispos..

[117] McCaffrey G., Staatz W.D., Sanchez-Covarrubias L., Finch J.D., DeMarco K., Laracuente M., Ronaldson P.T., Davis T.P. (2012). P-glycoprotein Trafficking at the Blood–brain Barrier Altered by Peripheral Inflammatory Hyperalgesia. J. Neurochem..

[118] Ossipov M.H., Kovelowski C.J., Porreca F. (1995). The Increase in Morphine Antinociceptive Potency Produced by Carrageenan-Induced Hindpaw Inflammation Is Blocked by Naltrindole, a Selective Δ-Opioid Antagonist. Neurosci. Lett..

[119] Cui Y.J., Cheng X., Weaver Y.M., Klaassen C.D. (2009). Tissue Distribution, Gender-Divergent Expression, Ontogeny, and Chemical Induction of Multidrug Resistance Transporter Genes (Mdr1a, Mdr1b, Mdr2) in Mice. Drug Metab. Dispos..

[120] Tome M.E., Herndon J.M., Schaefer C.P., Jacobs L.M., Zhang Y., Jarvis C.K., Davis T.P. (2016). P-Glycoprotein Traffics from the Nucleus to the Plasma Membrane in Rat Brain Endothelium during Inflammatory Pain. J. Cereb. Blood Flow Metab..

[121] Tome M.E., Jarvis C.K., Schaefer C.P., Jacobs L.M., Herndon J.M., Hunn K.C., Arkwright N.B., Kellohen K.L., Mierau P.C., Davis T.P. (2018). Acute pain alters P-glycoprotein-containing protein complexes in rat cerebral microvessels: Implications for P-glycoprotein trafficking. J. Cereb. Blood Flow Metab..

[122] Bendayan R., Ronaldson P.T., Gingras D., Bendayan M. (2006). In Situ Localization of P-Glycoprotein (ABCB1) in Human and Rat Brain. J. Histochem. Cytochem..

[123] Tome M.E., Schaefer C.P., Jacobs L.M., Zhang Y., Herndon J.M., Matty F.O., Davis T.P. (2015). Identification of P-glycoprotein Co-fractionating Proteins and Specific Binding Partners in Rat Brain Microvessels. J. Neurochem..

[124] Huber J.D., Witt K.A., Hom S., Egleton R.D., Mark K.S., Davis T.P. (2001). Inflammatory Pain Alters Blood-Brain Barrier Permeability and Tight Junctional Protein Expression. Am. J. Physiol. Heart Circ. Physiol..

[125] McCaffrey G., Willis C.L., Staatz W.D., Nametz N., Quigley C.A., Hom S., Lochhead J.J., Davis T.P. (2009). Occludin Oligomeric Assemblies at Tight Junctions of the Blood-Brain Barrier are Altered by Hypoxia and Reoxygenation Stress. J. Neurochem..

[126] Ronaldson P.T., DeMarco K.M., Sanchez-Covarrubias L., Solinsky C.M., Davis T.P. (2009). Transforming Growth Factor-Β Signaling Alters Substrate Permeability and Tight Junction Protein Expression at the Blood-Brain Barrier during Inflammatory Pain. J. Cereb. Blood Flow Metab..

[127] Gordh T., Sharma H.S. (2006). Chronic Spinal Nerve Ligation Induces Microvascular Permeability Disturbances, Astrocytic Reaction, and Structural Changes in the Rat Spinal Cord. Acta Neurochir. Suppl..

[128] Beggs S., Liu X.J., Kwan C., Salter M.W. (2010). Peripheral Nerve Injury and TRPV1-Expressing Primary Afferent C-Fibers Cause Opening of the Blood-Brain Barrier. Mol. Pain.

[129] Campos C.R., Ocheltree S.M., Hom S., Egleton R.D., Davis T.P. (2008). Nociceptive Inhibition Prevents Inflammatory Pain Induced Changes in the Blood–brain Barrier. Brain Res..

[130] Husain S., Abdul Y., Potter D.E. (2012). Non-analgesic effects of opioids: Neuroprotection in the retina. Curr. Pharm. Des..

[131] Tian X., Guo J., Zhu M., Li M., Wu G., Xia Y. (2013). δ-Opioid receptor activation rescues the functional TrkB receptor and protects the brain from ischemia-reperfusion injury in the rat. PLoS ONE.

[132] Crowley M.G., Liska M.G., Lippert T., Corey S., Borlongan C.V. (2017). Utilizing delta opioid receptors and peptides for cytoprotection: Implications in stroke and other neurological disorders. CNS Neurol. Disord. Drug Targets.

[133] Vaidya B., Sifat A.E., Karamyan V.T., Abbruscato T.J. (2018). The neuroprotective role of the brain opioid system in stroke injury. Drug Discov. Today.

[134] Yang L., Shah K., Wang H., Karamyan V.T., Abbruscato T.J. (2011). Characterization of neuroprotective effects of biphalin, an opioid receptor agonist, in a model of focal brain ischemia. J. Pharmacol. Exp. Ther..

[135] Yang L., Wang H., Shah K., Karamyan V.T., Abbruscato T.J. (2011). Opioid receptor agonists reduce brain edema in stroke. Brain Res..

[136] Yang L., Islam M.R., Karamyan V.T., Abbruscato T.J. (2015). In vitro and in vivo efficacy of a potent opioid receptor agonist, biphalin, compared to subtype-selective opioid receptor agonists for stroke treatment. Brain Res..

[137] Lee J.Y., Liska M.G., Crowley M., Xu K., Acosta S.A., Borlongan C.V., Guedes V.A. (2018). Multifaceted effects of delta opioid receptors and DADLE in diseases of the nervous system. Curr. Drug Discov. Technol..

[138] Islam M.R., Yang L., Lee Y.S., Hruby V.J., Karamyan V.T., Abbruscato T.J. (2016). Enkephalin-fentanyl multifunctional opioids as potential neuroprotectants for ischemic stroke treatment. Curr. Pharm. Des..

[139] Zohar O., Getslev V., Miller A.L., Schreiber S., Pick C.G. (2006). Morphine protects for head trauma induced cognitive deficits in mice. Neurosci. Lett..

[140] Lesniak A., Pick C.G., Misicka A., Lipkowski A.W., Sacharczuk M. (2016). Biphalin protects against cognitive deficits in a mouse model of mild traumatic brain injury (mTBI). Neuropharmacology.

[141] Statler K.D., Alexander H., Vagni V., Dixon C.E., Clark R.S., Jenkins L., Kochanek P.M. (2006). Comparison of seven anesthetic agents on outcome after experimental traumatic brain injury in adult, male rats. J. Neurotrauma.

[142] Ji B.S., Cen J., He L., Liu M., Liu Y.Q., Liu L. (2013). Modulation of P-glycoprotein in rat brain microvessel endothelial cells under oxygen glucose deprivation. J. Pharm. Pharmacol..

[143] Dazert P., Suofu Y., Grube M., Popa-Wagner A., Kroemer H.K., Jedlitschky G., Kessler C. (2006). Differential regulation of transport proteins in the periinfarct region following reversible middle cerebral artery occlusion in rats. Neuroscience.

[144] Spudich A., Kilic E., Xing H., Kilic U., Rentsch K.M., Wunderli-Allenspach H., Bassetti C.L., Hermann D.M. (2006). Inhibition of multidrug resistance transporter-1 facilitates neuroprotective therapies after focal cerebral ischemia. Nat. Neurosci..

[145] Cen J., Liu L., Li M.S., He L., Wang L.J., Liu Y.Q., Liu M., Ji B.S. (2013). Alteration in P-glycoprotein at the blood-brain barrier in the early period of MCAO in rats. J. Pharm. Pharmacol..

[146] DeMars K.M., Yang C., Hawkins K.E., McCrea A.O., Siwarski D.M., Candelario-Jalil E. (2017). Spatiotemporal changes in P-glycoprotein levels in brain and peripheral tissues following ischemic stroke in rats. J. Exp. Neurosci..

[147] Kilic E., Spudich A., Kilic U., Rentsch K.M., Vig R., Matter C.M., Wunderli-Allenspach H., Fritschy J.M., Bassetti C.L., Hermann D.M. (2008). ABCC1: A gateway for pharmacological compounds to the ischemic brain. Brain.

[148] Pop V., Sorensen D.W., Kamper J.E., Ajao D.O., Murphy M.P., Head E., Hartman R.E., Badaut J. (2013). Early brain injury alters the blood-brain barrier phenotype in parallel with β-amyloid and cognitive changes in adulthood. J. Cereb. Blood Flow Metab..

[149] Willyerd F.A., Empey P.E., Philbrick A., Ikonomovic M.D., Puccio A.M., Kochanek P.M., Okonkwo D.O., Clark R.S. (2016). Expression of ATP-binding cassette transporters B1 and C1 after severe traumatic brain injury in humans. J. Neurotrauma.

[150] Ibrahim M.M., Rude M.L., Stagg N.J., Mata H.P., Lai J., Vanderah T.W., Porreca F., Buckley N.E., Makriyannis A., Malan T.P. (2007). CB2 Cannabinoid Receptor Mediation of Antinociception. Pain.

[151] Zhang H., Lund D.M., Ciccone H.A., Staatz W.D., Ibrahim M.M., Largent-Milnes T.M., Seltzman H.H., Spigelman I., Vanderah T.W. (2018). Peripherally Restricted Cannabinoid 1 Receptor Agonist as a Novel Analgesic in Cancer-Induced Bone Pain. Pain.

[152] Ong C.K.S., Seymour R.A., Lirk P., Merry A.F. (2010). Combining Paracetamol (Acetaminophen) with Nonsteroidal Antiinflammatory Drugs: A Qualitative Systematic Review of Analgesic Efficacy for Acute Postoperative Pain. Anesth. Analg..

[153] Bannister K., Qu C., Navratilova E., Oyarzo J., Xie J.Y., King T., Dickenson A.H., Porreca F. (2017). Multiple Sites and Actions of Gabapentin-Induced Relief of Ongoing Experimental Neuropathic Pain. Pain.

[154] Serpell M., Latymer M., Almas M., Ortiz M., Parsons B., Prieto R. (2017). Neuropathic Pain Responds Better to Increased Doses of Pregabalin: An In-Depth Analysis of Flexible-Dose Clinical Trials. J. Pain Res..

[155] Godfrey L., Iannitelli A., Garrett N.L., Moger J., Imbert I., King T., Porreca F., Soundararajan R., Lalatsa A., Schätzlein A.G. (2018). Nanoparticulate Peptide Delivery Exclusively to the Brain Produces Tolerance Free Analgesia. J. Control. Release.

[156] Hayden J.A., van Tulder M.W., Tomlinson G. (2005). Systematic Review: Strategies for using Exercise Therapy to Improve Outcomes in Chronic Low Back Pain. Ann. Intern. Med..

[157] Ibrahim M.M., Patwardhan A., Gilbraith K.B., Moutal A., Yang X., Chew L.A., Largent-Milnes T., Malan T.P., Vanderah T.W., Porreca F. (2017). Long-Lasting Antinociceptive Effects of Green Light in Acute and Chronic Pain in Rats. Pain.

